# Molecular Mechanisms Underlying Accelerated Aging by Defects in the FGF23-Klotho System

**DOI:** 10.1155/2018/9679841

**Published:** 2018-05-21

**Authors:** Makoto Kuro-o

**Affiliations:** ^1^Division of Anti-Aging Medicine, Center for Molecular Medicine, Jichi Medical University, 3311-1 Yakushiji, Shimotsuke, Tochigi 329-0498, Japan; ^2^Department of Internal Medicine, Division of Mineral Metabolism, University of Texas Southwestern Medical Center, 5323 Harry Hines Blvd., Dallas, TX 75390-9072, USA

## Abstract

The basic research of aging has been primarily focused on elucidating mechanisms of aging and longevity that are evolutionarily conserved from yeasts to primates. Such efforts have culminated in the notion that (1) senescence at the cellular level is associated with aging at the organismal level and that (2) calorie restriction and growth suppression decelerate aging. However, these important findings in the basic research have not necessarily been linked to improvement of daily medical practice in the aging society. It has become increasingly important to investigate mechanisms of aging unique to mammals or humans and apply the research fruits for the treatment of major age-related disorders to extend the health span. Seminal studies on the* klotho* mouse, a mutant exhibiting a premature aging syndrome, have identified phosphate as a proaging factor in mammals. In this review, mechanisms of phosphate-induced premature aging and potential therapeutic targets will be discussed, which may be directly applicable for developing novel strategies for the treatment of chronic kidney disease and its complications.

## 1. Introduction

In 1997, a mutant mouse that displayed phenotypes resembling human premature aging syndromes was reported [1]. The aging-like phenotypes included shortened lifespan, atrophy of multiple organs (gonads, thymus, skin, etc.), vascular calcification, cardiac hypertrophy, osteopenia, sarcopenia, cognition impairment, hearing disturbance, and frailty. These symptoms were attributed to abolished expression of the* klotho* gene caused by an insertional mutation at its 5′ flanking region [1] associated with DNA hypermethylation [2]. The gene was named after a Greek goddess Klotho who spins the thread of life. The* klotho* gene encodes a single-pass transmembrane protein and is expressed only in a few tissues such as renal tubular cells [1] and parathyroid chief cells [3]. It was a new gene at that time and different from any genes defective in human premature aging syndromes that encode cytoplasmic or nuclear proteins involved in DNA repair.

In 2006, Klotho protein function was identified as a receptor for fibroblast growth factor-23 (FGF23) [4]. This discovery was prompted by the fact that* Fgf23*^−/−^ mice [5] and Klotho-deficient* (kl*/*kl)* mice exhibited identical phenotypes, namely, aging-like phenotypes associated with disturbed mineral metabolism. FGF23 is a peptide hormone secreted from osteocytes and/or osteoblasts upon phosphate intake. FGF23 then acts on the kidney to increase urinary phosphate excretion by suppressing phosphate reabsorption in renal proximal tubules, thereby maintaining the phosphate balance. FGF23 also suppresses synthesis of the active form of vitamin D (1,25-dihydroxyvitamin D_3_) in proximal tubules. Unlike the other members of the FGF family, FGF23 requires Klotho for binding and activating its cognate fibroblast growth factor receptors (FGFRs) [6].

Recently, the crystal structure of the Klotho-FGFR1c-FGF23 complex was resolved [7]. The extracellular domain of Klotho protein has a long arm that directly interacts with FGFR1c, termed the receptor-binding arm (RBA). By analogy from the protein structure of another member of the Klotho family (*β*Klotho) [8], the RBA of Klotho is supposed to have an intrinsically disordered structure and adopts a fixed configuration after binding to the extracellular domain of FGFR1c [9]. FGF23 fits into the groove generated between Klotho and FGFR1c. These findings have provided a structural basis for the unique feature that FGF23 requires Klotho to exert its hormonal actions.

## 2. Evolution of the* Klotho* Gene

During the evolution of life on earth, the first organisms that stored phosphorus in their body are bony fishes (Osteichthyes) that appeared in the Devonian period (~400 million years ago) [6, 10]. They accumulated phosphorus in the bone as calcium phosphate (CaPi). At that time, some other organisms such as sharks (Chondrichthyes) and shellfish (Mollusca) had skeletons made of cartilage and calcium carbonate. When compared with these skeletons, the bone made of CaPi or hydroxyapatite was much harder and stronger. It might be a prerequisite for the evolution of terrestrial animals to acquire hard and strong skeletons made of CaPi, which enable them to support their own body weight in the absence of buoyancy. In this context, it is interesting to note that orthologs of the* klotho* gene exist only in vertebrates harboring the bone made of CaPi. The* klotho* gene might have been evolved under the necessity in the regulation of phosphate metabolism.

## 3. Phosphate and Aging

The fact that FGF23 functions as a phosphaturic hormone as well as a counterregulatory hormone against vitamin D in a Klotho-dependent manner implies that the fundamental pathophysiology of mice lacking either FGF23 or Klotho is phosphate retention and vitamin D intoxication. In fact, these mice suffer from hyperphosphatemia and hypervitaminosis D [11]. Provided that the disturbed mineral metabolism is responsible for the aging-like phenotypes, these mice should be rescued by resolving phosphate retention and/or vitamin D intoxication.

Several laboratories tested this notion and identified interventions that rescue FGF23-deficient mice and/or Klotho-deficient mice from the aging-like symptoms [12]. Such interventions included feeding of vitamin D-deficient diet or low phosphate diet, ablation of the vitamin D receptor* (Vdr)* gene, the* Cyp27b1* gene, and the sodium-dependent phosphate transporter type IIa* (Npt2a)* gene (Table 1). The* Cyp27b1* gene encodes 1*α*-hydroxylase, the enzyme necessary for synthesis of 1,25-dihydroxyvitamin D_3_. The* Npt2a* gene is expressed in renal proximal tubules, where Npt2a protein is located on the apical brush border membrane and functions as the major entry gate of phosphate reabsorption from the tubular fluid in the process of transcellular phosphate transport. Among these interventions, vitamin D-deficient diet and ablation of the* Vdr* or* Cyp27b1* gene lowered both serum phosphate and 1,25-dihydroxyvitamin D_3_ levels. In contrast, low phosphate diet and ablation of the* Npt2a* gene lowered serum phosphate levels but increased 1,25-dihydroxyvitamin D_3_ levels, which is regarded as a compensatory attempt to maximize phosphate absorption from the diet. Despite the further increase in 1,25-dihydroxyvitamin D_3_, these interventions alleviated the aging-like phenotypes, indicating that it was not vitamin D but phosphate that was responsible for the premature aging syndrome. These observations have led us to conclude that phosphate accelerates aging [13]. Of note, association between accelerated aging and elevated serum phosphate levels is also observed in some patients with Hutchinson-Gilford syndrome, a rare hereditary progeroid disease caused by mutation in the lamin A gene, although high phosphate may not be the primary reason for their premature aging symptoms [14].

## 4. Phosphate Toxicity

It has been known in tissue culture experiments that extracellular phosphate is toxic to various types of cells at high concentration. For example, increase in the phosphate concentration in the tissue culture medium was reported to induce cell damage, apoptosis, and calcification in vascular endothelial cells and smooth muscle cells [15, 16]. The “phosphate toxicity” may contribute to the mechanism by which phosphate accelerates aging.

The phosphate toxicity observed in cultured cells was alleviated by adding phosphonoformic acid (PFA) to the high phosphate medium [16], which inhibits sodium-dependent phosphate cotransporter (Npt). Thus, it was thought necessary for phosphate to enter the cell to exert the cytotoxic effects. However, several lines of evidence have argued against this notion. First, the ability of PFA to inhibit phosphate-induced calcification of cultured vascular smooth muscle cells was shown independent of cellular phosphate uptake. Vascular smooth muscle cells express type III Npt involved in house-keeping cellular phosphate uptake, but not type II Npt involved in transepithelial phosphate resorption in renal proximal tubular cells. PFA inhibits type II Npt but barely inhibits type III Npt [17]. Instead, PFA inhibits formation of CaPi crystals like pyrophosphate and bisphosphonate, suggesting that the ability of PFA to inhibit calcification may be actually attributed to its ability to inhibit CaPi crystal formation [18]. Second, addition of insoluble CaPi crystals to the medium was shown to induce cellular damage and calcification [19, 20]. The concentration of calcium and phosphate ions in regular tissue culture medium is around 2 mM and 1 mM, respectively. Because this is a supersaturated condition regarding various types of CaPi precipitates, a small increase in the phosphate concentration can induce precipitation of CaPi. Therefore, the true culprit of phosphate toxicity may not be phosphate per se but CaPi crystals. Unfortunately, this possibility has not been explored in depth.

## 5. Calciprotein Particles (CPP)

Acquisition of the bone made of CaPi requires a defense mechanism that prevents CaPi from growing into large crystals in the extraosseous tissues and in the extracellular fluid. The extracellular fluid in mammals is supersaturated in terms of calcium and phosphate ions and therefore at high risk for CaPi precipitation. However, CaPi crystals never grow in the blood, because serum protein fetuin-A absorbs tiny CaPi precipitates and prevents them from growing into large crystals. As a result, aggregates of CaPi-laden fetuin-A molecules are generated and dispersed in the blood as colloidal particles, which are called calciprotein particles (CPP) [21]. Thus, CaPi in the blood exist in the form of CPP.

Although less potent than CaPi, CPP can also induce calcification in vascular smooth muscle cells [22] and innate immune responses in macrophages in culture [23]. Recent clinical studies have indicated that serum CPP levels are increased with decline of renal function and associated with clinical parameters for vascular stiffness, vascular calcification, and noninfectious chronic inflammation [24, 25]. These observations have raised the possibility that the relation between CPP and these pathological conditions may not merely be an association but a causation. Namely, the ability of CPP to behave like a “pathogen” may contribute to arteriosclerosis and chronic inflammation in patients with chronic kidney disease (CKD), a clinical model for accelerated aging [26].

Formation of CPP is primarily a physicochemical process affected not only by concentration of calcium and phosphate ions but also by various factors such as pH, temperature, ionic strength, incubation time, composition, and concentration of other ions and proteins in the solution. Depending on these parameters, physical properties of CPP, including particle size distribution, protein-mineral ratio, and the surface charge (zeta potential), and the CaPi phase (amorphous or crystalline) should vary. In fact, when formation of CPP was induced in test tubes by adding calcium (10 mM) and phosphate (6 mM) to serum, physical properties of CPP changed dynamically over time [27]. Spheroidal CPP carrying amorphous CaPi were generated within an hour, which were smaller than 100 nm in diameter and called primary CPP. Thereafter, a sudden increase in turbidity was observed due to the Tyndall phenomenon caused by formation of ellipsoidal CPP carrying crystalline CaPi, which were larger than primary CPP and called secondary CPP. Transition from primary CPP to secondary CPP occurred spontaneously within a few hours after addition of calcium and phosphate. The time required for formation of secondary CPP serves as a marker for the serum propensity for CPP formation. The shorter the time, the higher the serum propensity for CPP formation. A clinical study using stage 3 and 4 CKD patients showed that the serum propensity for CPP formation was associated with vascular stiffness and all-cause mortality [25, 28]. Thus, this assay is thought useful not only as a clinical test for evaluation of the risk for arteriosclerosis but also as a support of the notion that CPP may contribute to acceleration of CKD complications and perhaps aging.

## 6. CPP Levels in the Blood

Several different methods have been reported for quantification of CPP levels in the blood. Based on the assumption that CPP are precipitated by centrifugation at 16,000~30,000*g* for 120 minutes, difference in the serum fetuin-A levels between before and after the centrifugation has been used as a surrogate of serum CPP levels [24, 25]. Thereafter, novel CPP assays were reported that directly detected CPP in the serum/plasma using a fluorescent probe for CaPi crystals [29, 30]. Of note, one of these studies identified low-density CPP that were not precipitated by the centrifugation and demonstrated that serum phosphate and age were the two major independent predictor variables of plasma CPP levels in the population without hyperphosphatemia [30]. Because serum phosphate levels, even within the normal range, were reported to correlate with all-cause mortality [31], it is intriguing to speculate that high blood phosphate is a risk for high blood CPP that may accelerate aging and increase all-cause mortality through inducing chronic noninfectious inflammation. In support of this speculation, serum phosphate levels inversely correlate with longevity in mammals [12].

## 7. CPP in the Urine

Renal toxicity of dietary phosphate load was reported several decades ago [32]. In that report, normal rats and uninephrectomized rats were placed on diet containing various amount of phosphate for 18 weeks and then evaluated for renal histology. The conclusion was that phosphate excretion per nephron, but not serum phosphate, was correlated with a score of histological changes that reflected severity of tubular damage and interstitial fibrosis. Although the FGF23-Klotho endocrine system was not known at that time, if serum FGF23 had been measured, it should have been positively correlated with phosphate excretion per nephron. Because FGF23 suppresses phosphate resorption at the proximal tubules, FGF23 should increase phosphate concentration and thus the risk for formation of CaPi in the proximal tubular fluid. Indeed, micropuncture studies indicated that concentration of calcium and phosphate ions in the proximal tubular fluid could exceed the solubility limit upon dietary phosphate load [33]. Although it remains to be determined whether CPP indeed appear in the tubular lumen, it is possible that high FGF23 that increases phosphate excretion per nephron may induce tubular damage and renal fibrosis by promoting CPP formation in the tubular fluid, regardless of serum phosphate levels.

## 8. Phosphate Restriction in Future

Since hyperphosphatemia was identified as a risk for vascular calcification and cardiovascular events, phosphate restriction by means of diet counseling and administration of phosphate binders has been justified for CKD patients with hyperphosphatemia [34–36]. The goal of phosphate restriction is to reduce serum phosphate levels. However, it should be noted that hyperphosphatemia is a terminal symptom during CKD progression observed in patients with end-stage renal disease (ESRD), whose residual nephron number is estimated as 1/10 or less of normal individuals [37]. In fact, phosphate binders are prescribed mostly for dialysis patients. Although phosphate binders are expected to improve prognosis of CKD patients, a recent network meta-analysis study failed to show evidence that phosphate binder treatment reduced mortality compared with placebo in ESRD patients [38]. It may be necessary to reevaluate the current paradigm for phosphate restriction.

Because increase in phosphate excretion per nephron can induce tubular and interstitial damage, it is reasonable to start phosphate restriction when phosphate excretion per nephron is increased. Because FGF23 is a phosphaturic hormone that increases phosphate excretion per nephron, FGF23 likely serves as a surrogate marker for phosphate excretion per nephron. Thus, increase in FGF23 should be recognized as a sign of excess phosphate intake relative to the residual nephron number and a ground for starting phosphate restriction regardless of serum phosphate levels. Because FGF23 increases long before hyperphosphatemia ensues during CKD progression [39], phosphate binders should be started at stages 2 or 3 when serum FGF23 levels, but not serum phosphate levels, start increasing. In this new paradigm for phosphate restriction, the goal is to lower serum FGF23 levels, but not serum phosphate levels. The outcome to be evaluated must be tubular damage, but not vascular calcification, cardiovascular events, or mortality (Table 2). This new paradigm requires justification by clinical studies.

## 9. Conclusion

Discovery of the* klotho* gene and its function as the obligate coreceptor for FGF23 revealed a novel endocrine axis indispensable for maintaining phosphate homeostasis. The fact that Klotho- or FGF23-deficient mice exhibit premature aging and are restored from the aging-like phenotypes by dietary phosphate restriction leads to the notion that phosphate accelerates aging. Phosphate exerts its toxicity when it binds to calcium and becomes CaPi. In the blood, CaPi binds to serum protein fetuin-A and forms colloidal particles termed CPP. CPP increase with age and serum phosphate levels. CPP can induce vascular endothelial damage, smooth muscle calcification, and innate immune responses, leading to arteriosclerosis and chronic noninfectious inflammation. In the renal tubular fluid, CPP may appear when phosphate excretion per nephron is increased by FGF23. CPP induce renal tubular damage and interstitial fibrosis to accelerate kidney aging and exacerbate CKD. Provided that CPP, but not phosphate per se, are responsible for phosphate toxicity and thus accelerate aging, CPP may be justified as a novel therapeutic target for aging and age-related disorders.

## Figures and Tables

**Table 1:** Interventions that alleviate the aging-like phenotypes in Klotho-deficient mice and/or FGF23-deficient mice and associated changes in serum levels of phosphate and 1,25-dihydroxyvitamin D_3_ (Vitamin D).

Interventions	Changes in serum levels	
	Phosphate	Vitamin D
Vitamin D-deficient diet	↓	↓
Ablation of vitamin D receptor	↓	↓
Ablation of 1*α*-hydroxylase	↓	↓
Ablation of Npt2a	↓	↑
Low phosphate diet	↓	↑

**Table 2:** Comparison between the current paradigm and proposed paradigm for phosphate restriction.

	Current paradigm	Proposed paradigm
Rationale	Hyperphosphatemia is a risk for vascular calcification and mortality	Increase in phosphate excretion per nephron is a risk for renal tubular damage
Goal	To lower serum phosphate levels	To lower serum FGF23 levels
Outcome	Vascular calcification, cardiovascular events, death	Renal tubular damage
Indication	Hyperphosphatemia (~0.35% of the total CKD patients)	Hyper-FGF23-emia (~8% of the total CKD patients)

## Abbreviations

FGF23: Fibroblast growth factor-23
FGFR: Fibroblast growth factor receptor
Npt2a: Sodium-dependent phosphate transporter type IIa
Npt3: Sodium-dependent phosphate transporter type III
PFA: Phosphonoformic acid
Vdr: Vitamin D receptor
Cyp27b1: Cytochrome P450 family 27 subfamily B member 1
CaPi: Calcium phosphate
CPP: Calciprotein particles
CKD: Chronic kidney disease.
